# A Case of Localized Pulmonary Calcification Presenting as a Persistent Mass Lesion in an Immunosuppressed Patient Following Treatment of a Pseudomonas Pneumonia

**DOI:** 10.7759/cureus.1765

**Published:** 2017-10-11

**Authors:** Matthew J Cecchini, Dominic L Shepherd, Jessica G Shepherd

**Affiliations:** 1 Department of Pathology, Schulich School of Medicine & Dentistry, Western University, London, Ontario, CA; 2 Schulich School of Medicine & Dentistry, Western University, London, Ontario, CA; 3 Department of Pathology and Laboratory Medicine, London Health Science Centre

**Keywords:** pulmonary calcification, immunosupressed, pseudomonas

## Abstract

We report a case of a persistent right upper lobe opacity following treatment for a *Pseudomonas* infection in an immunosuppressed patient with a recent renal transplantation. The patient underwent a surgical lung biopsy for definitive diagnosis of the mass. The lesion was composed of extensive calcifications deposited throughout the lung with associated fibrosis. The patient had a history of a remote parathyroidectomy for hyperparathyroidism; however, the parathyroid hormone (PTH) and the calcium levels were still mildly elevated. No other calcified lung lesions had developed in a follow-up after the initial resection. Pulmonary calcification has been classically associated with varicella pneumonia; no viral cytopathic changes were identified for varicella or other viruses in this case. The calcification appears to be secondary to the recent *Pseudomonas* pneumonia. To our knowledge, this is the first report of a *Pseudomonas* pneumonia resulting in extensive localized pulmonary calcification. This is an important diagnostic consideration as this benign entity should be considered in patients with persistent opacities following treatment for pneumonia.

## Introduction

Parenchymal pulmonary calcification can be separated into dystrophic and metastatic calcifications. Dystrophic calcification occurs in damaged lungs typically following an inflammatory process as a localized organized collection of hydroxyapatite calcium salt [1]. Metastatic pulmonary calcification is caused by high serum calcium and phosphate levels that accumulate in normal lung tissue. The elevated calcium is usually from renal failure, hyperparathyroidism or neoplastic destruction of bone [1]. Calcium deposition favours alkaline tissues, in particular in the upper lobes of the lungs, which have a high ventilation to perfusion (V/Q) ratio and low end capillary pCO_2_, resulting in a more alkaline pH [2-3]. Pulmonary calcifications are typically incidental findings identified on imaging and rarely result in significant respiratory disease [1].

We report a case of a patient presenting with a persistent right upper lobe mass following a *Pseudomonas* pneumonia. There was a history of chronic kidney disease with renal transplantation and a remote history of a parathyroidectomy with residual mildly elevated parathyroid hormone (PTH) and calcium levels.

## Case presentation

**Figure 1:**
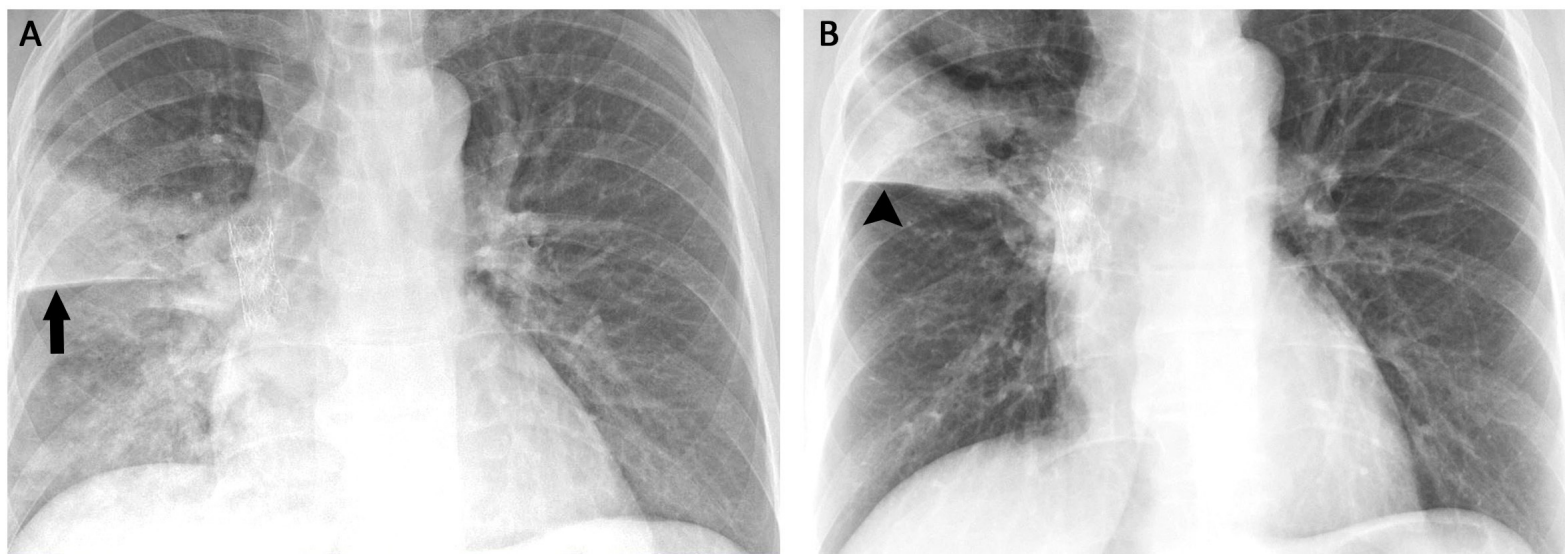
(A) Chest radiograph demonstrating the right upper lobe pneumonia (arrow) and (B) persistent right upper lobe opacity (arrowhead) following treatment of the pneumonia.

**Figure 2:**
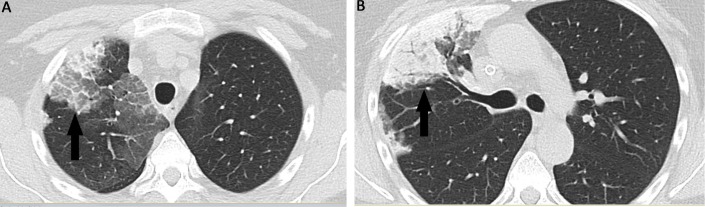
(A, B) Representative CT slices showing the right upper lobe opacity (arrow). CT - computed tomography.

The patient is a female in her 40s who underwent a repeat renal transplantation that was complicated by acute tubular necrosis, delayed graft function, and *Pseudomonas* sepsis with a right upper lobe pneumonia (Figure 1A). The patient was admitted and treated with an extended course of piperacillin-tazobactam for the *Pseudomonas* infection. She continued to experience dyspnea following the completion of treatment and had a persistent right upper lobe opacity identified on chest X-ray (Figure 1B) and the subsequent computed tomography (CT) scan (Figures 2A-2B). CT scans demonstrated a large opacity (10 cm x 4 cm) with adjacent areas of mixed ground glass opacity, airspace opacity, and thickening of the interlobular septa. The combination of the ground-glass attenuation with superimposed interlobular septal thickening and reticular thickening-specific opacifications was consistent with a “crazy paving” pattern. Given the history of immunosuppression, infectious, inflammatory, and neoplastic lesions were considered in the differential. A follow-up CT scan two months later demonstrated a persistent lesion with little change from the previous imaging. Given the failure of this lesion to resolve, the patient underwent a surgical lung biopsy to exclude an atypical infectious or neoplastic lesion.

**Figure 3:**
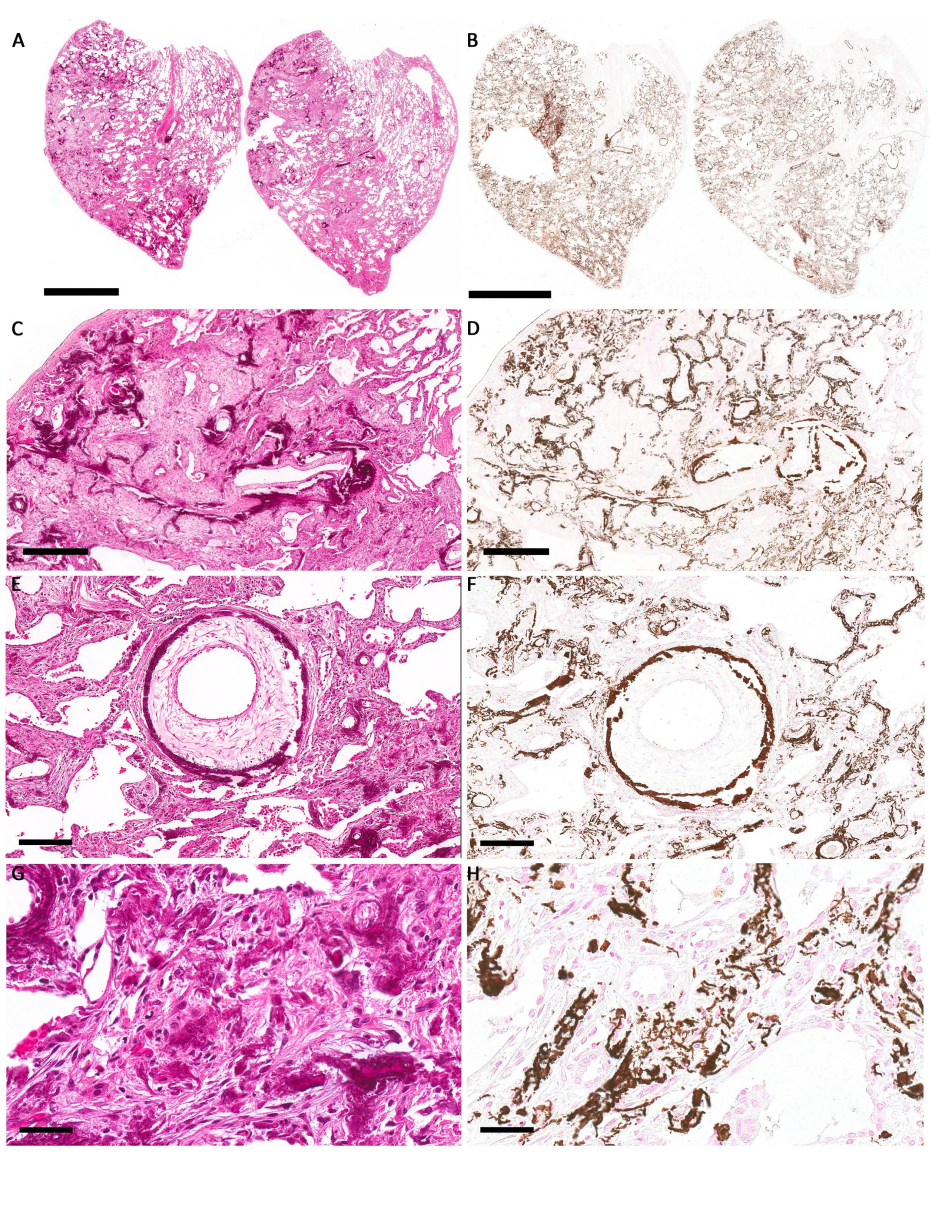
Photomicrographs of hematoxylin and eosin stain sections of the wedge biopsy (A, C, E, G) with corresponding areas stained with von Kossa histochemical stain (B, D, G, H) to highlight calcium deposition. (A, B) scale bar = 3 mm, (C, D) scale bar = 500 microns, (E, F) scale bar = 200 microns, (G, H) scale bar = 100 microns.

A video-assisted thoracoscopic surgery (VATS) was performed and an abnormal right upper lobe with a nodular and cheesy appearance was identified. A representative wedge biopsy was sent for intraoperative consultation. The cryosections showed interstitial pneumonitis with calcification and no evidence of malignancy. Given that this appeared to be a benign process, a lobectomy was not performed, but additional wedge biopsies were sent for permanent sections along with material for culture. As shown in Figures 3A, 3C, 3E, 3G, sections of the lung biopsy were composed of extensive multifocal calcifications, highlighted by a von Kossa stain (Figures 3B, 3D, 3F, 3H) . The calcifications ranged in size from small stippled foci to larger irregular deposits with scattered foreign body giant cell reactions. The calcifications were localized within the alveolar septa and were associated with collagenous fibrosis and focal fibroblastic proliferation (Figures 3G-3H). Many of the small blood vessels showed marked subendothelial myxoid intimal thickening with associated calcifications (Figures 3E-3F). In contrast, the larger veins and arteries were relatively spared from calcification. The cultures were negative and Grocott's methenamine silver stain (GMS) and Ziehl-Neelsen (ZN) stains on tissue sections were negative for fungal organisms and acid-fast bacteria, respectively.

**Figure 4:**
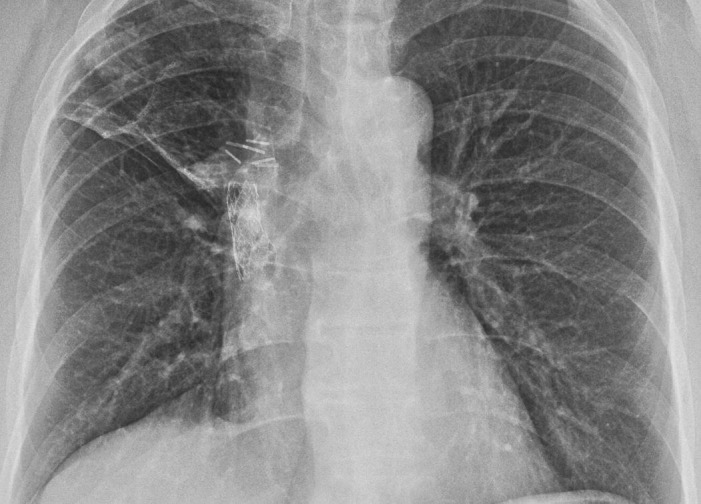
Follow up chest X-ray after surgical lung biopsy showing resolution of the right upper lobe opacity.

Following the biopsy, the PTH was found to be mildly elevated at 8.1 pmol/L and associated with a slightly elevated calcium of up to 2.7 mmol/L. In a follow-up six months after the resection, the patient had normal pulmonary function testing, and the X-ray shown in Figure 4 showed a significantly reduced opacity in the right upper lobe of the lung with no new consolidations or lesions.

## Discussion

Pulmonary calcification is typically divided into dystrophic and metastatic calcification. This case represents an overlap of dystrophic and metastatic calcification. While the patient had an elevated serum calcium level, they also had a right upper lobe *Pseudomonas* pneumonia that may have acted as a nidus for a dystrophic type of calcification. Varicella pneumonia has been classically associated with pulmonary calcification [4]. There was no history of recent varicella infection and no viral cytopathic effects were identified on biopsy. Pulmonary calcification after resolution of other infections have been reported, including *Pneumocystis* and small pox [4-5]. However, to our knowledge there are no reports of pulmonary calcification following resolution of a *Pseudomonas* infection. Pulmonary calcifications can also be seen in patients with chronic kidney disease on dialysis [6]; however, these calcifications tend to be more diffuse in nature.

Prominent myxoid intimal thickening was observed in many of the calcified smaller vessels within the biopsy (Figures 3E-3F). This has been described as one of the earlier histologic features of pulmonary veno-occlusive disease [7], and recent infection has been suggested to be a potential causative agent [8]. Further, localized pulmonary calcification has been reported in association with vascular obstruction [9]. The mechanism is thought to involve a ventilation perfusion mismatch, where CO_2_ is still expelled via ventilation but the decreased perfusion results in a decreased supply of CO_2_ and an increase in the pH. This more alkaline pH can promote the deposition of calcium into the lung parenchyma.

While pulmonary calcifications tend to have a benign course, there are a number of renal transplantation cases with extensive pulmonary calcification that have resulted in acute respiratory failure and even death in isolated cases [10]. These cases have all been associated with kidney graft failure, and improvement in calcification has been seen with improvement of graft function and treatment of underlying hyperparathyroidism [10]. In this case, the patient had a transient acute tubular necrosis that was associated with the underlying *Pseudomonas* sepsis that may have led to transient deterioration in graft function, which may have supported the development of calcification of the resolving right upper lobe pneumonia.

## Conclusions

We present a case of extensive localized pulmonary calcifications in an immunosuppressed patient with a recent kidney transplant and treatment for a *Pseudomonas* pneumonia. Given that the localization of the pneumonia appears to overlap with the area of pulmonary calcification, it is most likely that pulmonary calcification is related to the resolving post infectious process. This is classically reported in varicella pneumonia but has yet to be associated with a *Pseudomonas* infection. Further supporting this, the patient has not experienced any recurrence or involvement of other areas of the lung. Localized pulmonary calcification should be considered as part of the differential diagnosis in patients with persistent opacities following resolution of a pneumonia.
